# Synthesis and Optimization of Chitosan Nanoparticles Loaded with l-Ascorbic Acid and Thymoquinone

**DOI:** 10.3390/nano8110920

**Published:** 2018-11-07

**Authors:** Nurhanisah Othman, Mas Jaffri Masarudin, Cha Yee Kuen, Nurul Azira Dasuan, Luqman Chuah Abdullah, Siti Nurul Ain Md. Jamil

**Affiliations:** 1 Department of Chemistry, Faculty of Science, Universiti Putra Malaysia, Serdang 43400, Selangor, Malaysia; hanisahlab@gmail.com (N.O.); nurulzira95@gmail.com (N.A.D.); 2 Department of Cell and Molecular Biology, Faculty of Biotechnology and Biomolecular Sciences, Universiti Putra Malaysia, Serdang 43400, Selangor, Malaysia; masjaffri@upm.edu.my (M.J.M.); yeekuen_91@hotmail.com (C.Y.K.); 3 Cancer Research Laboratory, Institute of Biosciences, Universiti Putra Malaysia, Serdang 43400, Selangor, Malaysia; 4 Department of Chemical and Environmental Engineering, Faculty of Engineering, Universiti Putra Malaysia, Serdang 43400, Selangor, Malaysia; chuah@upm.edu.my

**Keywords:** antioxidant, chitosan, hydrophobic-hydrophilic, ionic gelation, l-ascorbic acid, nanoparticles, thymoquinone

## Abstract

The combination of compounds with different classes (hydrophobic and hydrophilic characters) in single chitosan carrier is a challenge due to the hydrophilicity of chitosan. Utilization of l-ascorbic acid (LAA) and thymoquinone (TQ) compounds as effective antioxidants is marred by poor bioavailability and uptake. Nanoparticles (NPs) solved the problem by functioning as a carrier for them because they have high surface areas for more efficient delivery and uptake by cells. This research, therefore, synthesized chitosan NPs (CNPs) containing LAA and TQ, CNP-LAA-TQ via ionic gelation routes as the preparation is non-toxic. They were characterized using electron microscopy, zetasizer, UV–VIS spectrophotometry, and infrared spectroscopy. The optimum CNP-LAA-TQ size produced was 141.5 ± 7.8 nm, with a polydispersity index (PDI) of 0.207 ± 0.013. The encapsulation efficiency of CNP-LAA-TQ was 22.8 ± 3.2% for LAA and 35.6 ± 3.6% for TQ. Combined hydrophilic LAA and hydrophobic TQ proved that a myriad of highly efficacious compounds with poor systemic uptake could be encapsulated together in NP systems to increase their pharmaceutical efficiency, indirectly contributing to the advancement of medical and pharmaceutical sectors.

## 1. Introduction 

Fighting infections or diseases may be difficult as a result of immunodeficiency disorders, which are caused by malnutrition [1]. Consequently, a number of research works have been published, reporting the initiatives done by researchers to provide nutritional supplements in many forms; one of them is through the development of NPs [2,3,4,5,6]. Nano-sized particles are capable to work as pharmaceutical carriers for various delivery system; orally, transdermally, or intravenously. Studies show that NPs have been tremendously used in the biomedical sector to treat diseases like diabetes [7,8,9] and cancer [10,11,12]. Additionally, particle size plays an important role in controlling the efficiency of transporting therapeutic agents by conventional ways. NPs are favored to deliver the medicine because their size is small and they have high surface area [13] for better pharmaceutical release to the target organ. Stage alteration of the polymer and encapsulation are needed to keep on producing better therapeutic delivery systems.

However, in innovating the transport system for therapeutic agents, many challenges need to be solved; one of them is the selection of nanomaterial. NPs can be made up of a list of materials but some existing NPs may be harmful to humans as they may generate toxicity to the body and environment during their production, application, and disposal. Some materials for NPs formation, mainly metal based are reported to cause undesirable side effects to living organisms. For instance, overconsumption of silver can cause argyria [14,15]. Sea urchin *Paracentrotus lividus* showed total mortality after two days of being exposed to 10 mg/L of tin oxide, cerium oxide, and iron oxide NPs [16]. Among all the existing NPs, a polymer that makes good NPs is chitosan (CS) due to its special properties. It has antimicrobial characteristics and is capable of healing wounds [17]. Additionally, CS is a biodegradable and biocompatible carbohydrate [10]. CS has also been widely used in the medical sector, for instance, in enhancing delivery of a cancer drug, silibinin [18]. This was achieved by functionalizing CS hydrophobically, enabling it to carry and release hydrophobic drugs with ease. CS is a highly modifiable polymer that can grafted or crosslinked to produce derivatives [19,20]. The presence of amino and hydroxyl groups in CS makes it a highly modifiable element for numerous study field [21]. Chitosan nanoparticles (CNPs) were previously found to be toxic to A549 lung cancer cells [18], but non-toxic to normal cells [22]; this demonstrates inherent anticancer properties.

CNPs are usually made up by ionic gelation method, which describes the crosslinking reaction of CS with TPP [23,24]. Positively charged CS interacts with the negatively-charged crosslinker called sodium tripolyphosphate (TPP) [25]. It was reported in a study that CS (0.1%, 0.2%, 0.3%) was mixed with TPP (0.02%, 0.05%, 0.08%) at room temperature to make CNPs. They worked on embedding a drug called propranolol into the NPs; the drug was mixed with CS solution first before adding the TPP solution, prior to the formation of NPs [26].

In another study, ionic gelation of CS with TPP was also implemented, but with alterations to increase stability and produce monodisperse, low molecular weight CNPs. Through optimization processes, NPs with smaller diameters (~138 nm) and good storage stability at room temperature were successfully produced. Major optimization focused on lowering acetic acid concentration and ambient temperature. However, many other parameters were considered: concentration of both CS and TPP solution, pH, and temperature of the CS solution and stirring speed. Ambient temperature during cross-linking plays an important role in forming high-quality NPs. Low ambient temperature was preferred because it causes a faster cooling rate of suspension, providing more hydrogen bond interaction between CS and water molecules. Not only that, at low ambient temperature CS molecules stiffen faster and eventually stabilized the particles’ structure [27].

As a carrier for pharmaceutical agents, NPs need to be encapsulated or embedded with compounds. In selecting compounds, these physicochemical properties have to be considered: molecular weight (<1000 Daltons), affinity for both lipophilic and hydrophilic phases, low melting point, short half-life, and non-irritating [28]. A compound consists of an enediol moiety, namely l-ascorbic acid (LAA), possesses a range of benefits, especially as a powerful antioxidant [29]. It has been used in CNPs to study the factors affecting shelf life and delivery enhancement. It was found that low molecular weight CS encapsulated LAA more efficiently. Additionally, the release of LAA from NPs depended on pH; a higher release rate could be observed when phosphate buffer solution (pH 7.4) was used, as compared to the low pH of 0.1 M HCl [30].

Another potential compound that was encapsulated in CNPs is also an antioxidant, namely thymoquinone, TQ; the main constituent in black seed herb. A research about TQ loaded CNPs, CNP-TQ was conducted for nose-to-brain targeting to indirectly treat Alzheimer’s disease. Encapsulation of 4.275 mg of TQ resulted in 0.130 polydispersity index (PDI) value with a diameter of 199.4 nm. Through the study, intranasal CNP-TQ was proven to be more effective in brain targeting as compared to TQ solution delivered by either intravenous or intranasal route [31].

Dual encapsulation had been proven to increase drugs release through a study of poly(lactic-co-glycolic acid) (PLGA) NPs encapsulated with anticancer drug, paclitaxel (PTX), and TQ. It used 10:1 ratio of polymer to drug; 400 mg of PLGA polymer with 25 mg of anticancer drug, paclitaxel (PTX) and 15 mg TQ to form PTX + TQ-loaded NPs [32]. By combining both drugs in NPs, they observed 19% and 5% increment of PTX and TQ release, respectively. This indicated that drugs released more efficiently from PTX + TQ-loaded NPs than single drug-loaded NPs [32].

**Figure 1:**
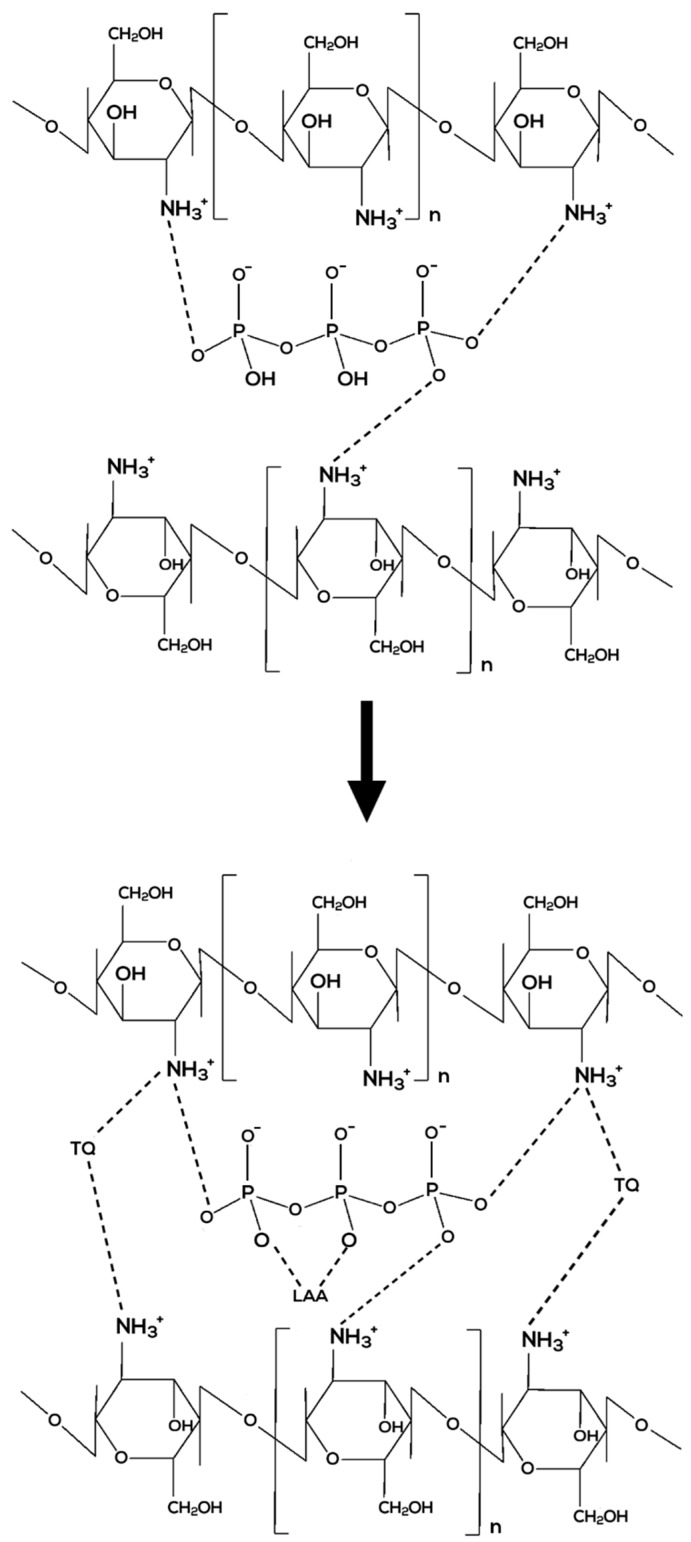
Proposed ionic interaction of CNP (**above**) and CNP-LAA-TQ (**below**).

In another aspect, encapsulating more than one compound together into CNPs may be challenging as they might not be compatible and may cause complications. However, in this project, two compounds that have proven their compatibility will be inserted; LAA and TQ. They make a good combination and produced synergistic effects by being able to treat seizures in rat brains. Research reported by Ullah et al. in 2014 suggested the compatibility between TQ and LAA in treating pentylenetetrazole (PTZ)-induced generalized seizures. PTZ is a drug used to study seizures that also acts as a gamma-aminobutyric acid (GABAA) receptor antagonist. In their study, treatment in six rat groups showed that TQ and LAA delayed the onset of PTZ-induced seizures, decreased high-grade seizures, and reduced the number of mortalities among PTZ-treated rats. TQ and LAA that were administered orally had synergistically activated expression of the GABAB1 receptor (stimulate K^+^ channel opening for neuron to achieve closer equilibrium potential of K^+^) and increased the expression of a Ca^2+^-activated enzyme called calcium/calmodulin-dependent protein kinase II (CaMKII). The study suggested that TQ and LAA possessed anticonvulsant and neuroprotective properties that are useful in treating epileptic seizures. This also indicates that TQ and LAA are compatible and collaboratively produced synergistic effects [33]. Therefore, in this study, LAA and TQ will be encapsulated in a CS carrier to form CNP-LAA-TQ as proposed in Figure 1.

**Figure 2:**
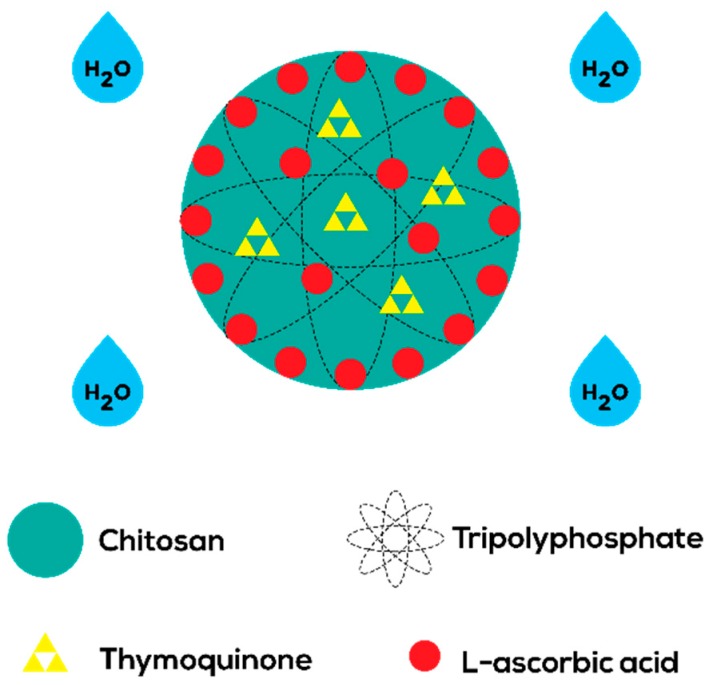
Proposed schematic illustration of CNP-LAA-TQ. Hydrophilic LAA binds to the surface and scatters around CNPs, while hydrophobic TQ stays inside the CNPs. This is due to the presence of hydrophilic water that lingers around the CNPs.

The encapsulation of both LAA and TQ is proposed in Figure 2. By creating this dual-class compound carrier, a range of illnesses that require combination compounds could be treated as it may be more potent than a single-compound carrier. Interaction between combined compounds may happen in two ways: (a) action augmentation of one agent by another agent, or (b) emergence of new effects as a result of the combination [34]. Cancer treatment often requires the right drug combinations to lessen tumor expansion and mobility. For instance, co-administration of hydrophobic curcumin (CUR) with hydrophilic doxorubicin (DOX) in LipoNiosome NPs with higher entrapment efficiency ~80% elevated the synergistic interaction between dual drugs as compared to single-drug systems [35]. The same goes to treating tuberculosis (TB); drug co-encapsulation in Brij-96 microemulsions resulted in the stabilization of rifampicin (RIF) in the presence of isoniazid (INH) and pyrazinamide (PZA). The combined drugs showed better antimicrobial activities compared to single-drug action [36].

Not only compatible together, LAA and TQ are both antioxidant agents [37,38]. Imbalance of free radicals and antioxidants results in a condition called oxidative stress, which triggers diseases. Therefore, an adequate amount of antioxidant is needed to control the level of reactive oxygen species (ROS). The presence of phenolic functional groups in TQ contributed to its selection as a pharmaceutical compound in this research. Examples of antioxidant activities of phenolics are the inhibition of ROS formation and entrapment of ROS. In short, CNP-LAA-TQ has the potential to aid the process of scavenging ROS to keep the body in good health. 

Consequently, this study aims for synthesizing compact CNPs with high encapsulation efficiency of both LAA and TQ. In order to achieve this, a list of essential parameters—amount of acetic acid, ambient temperature, pH of solutions, and centrifugation time and speed—are needed to be adjusted [39]. The optimization process can be controlled by performing a few characterization studies: Fourier transform infrared (FTIR), particle size distribution (PSD), encapsulation efficiency (EE) of LAA and TQ, and field-emission scanning electron microscopy (FESEM). The level of sizing correlates to different TPP concentrations to indicate the optimal parameters; for example, the smallest particle size and low PDI. Thus, it does not correlate to cellular experiments. 

## 2. Materials and Methods 

Chitosan (CS) with MW = 50,000–190,000 Da was used as carrier and sodium tripolyphosphate (TPP) with MW= 367.86 Da was used as crosslinking agent, both were purchased from Sigma-Aldrich (St. Louis, MO, USA). Glacial acetic acid, sodium hydroxide pallets, 5% *w*/*v* hydrochloric acid, l-ascorbic acid, and dimethylsulfoxide (DMSO) were purchased from R and M (UK). Thymoquinone as hydrophobic component was purchased from Sigma Aldrich (St. Louis, MO, USA). All chemicals were of analytical grade and used without any further purification. 

### 2.1. Preparation of CNP Samples

CNP was prepared by using ionic gelation method as reported by Masarudin et al. [40]. Firstly, ~1.0 mL of 1.0% acetic acid was added to low molecular weight CS to dissolve it and then further diluted to form a concentration of 0.5 mg/mL CS solution, CS. Next, the pH of the CS was adjusted to 5.0 by adding 1 M aqueous sodium hydroxide solution. The crosslinker, TPP was dissolved in distilled water to make a concentration of 0.7 mg/mL, which then altered to pH 2.0 by using 1.0 M hydrochloric acid. Primarily, CNP was formed by adding 600 μL of CS to a range volume of TPP solution (0 to 300 μL). The optimum volume of TPP solution that met the targeted size and other characteristics is explained in the Results and Discussion section. The mixture was then centrifuged at 13,000 rpm for 20 min to purify it. 

CNP-LAA is a version of single-loaded CNP, which was prepared by first pouring approximately 10.0 mg of LAA into 10.0 mL of 0.7 mg/mL TPP solution, making a LAA stock concentration of 5.7 mM. It was further diluted to three different intermediate concentrations. Then, 250 μL of each diluted LAA was added into three different tubes of 600 μL of 0.5 mg/mL CS solutions, to produce CNP-LAA. Final LAA concentrations used were 160, 235 and 310 μM. 

Another single-loaded CNP, CNP-TQ was prepared by dissolving approximately 2.0 mg of TQ in 2.0 mL of 99.0% of DMSO, making a stock concentration of 6.1 mM. It was further diluted to three different intermediate concentrations. Only 100 μL of each diluted TQ was added to three different tubes of 600 μL of 0.5 mg/mL of CS. Lastly, 250 μL of 0.7 mg/mL of TPP was added to produce CNP-TQ. Final TQ concentrations used were 100, 150, and 200 μM 

**Figure 3:**
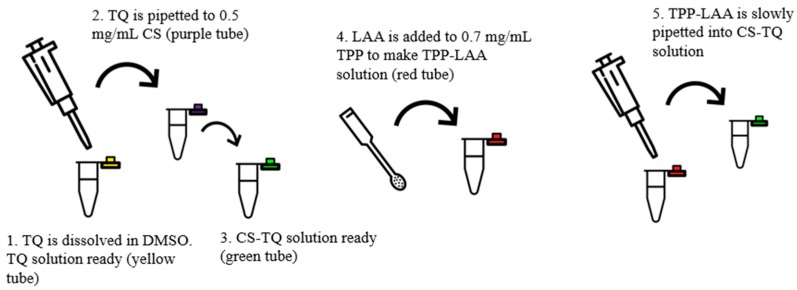
Illustration for dual encapsulation of LAA and TQ into CNPs.

The encapsulation of both compounds, LAA and TQ to make CNP-LAA-TQ is shown in Figure 3. The same TQ and LAA stock solution was prepared as above. A set of TQ concentrations (100, 150, and 200 μM) with a set of LAA concentrations (160, 235, and 310 μM) were tested to find the optimum formulation. About 100 μL of diluted TQ solution was added to 600 μL of 0.5 mg/mL of CS; the solution was mixed for a while. Then, 250 μL of LAA-diluted solution was poured into the mixture of TQ and CS.

### 2.2. Physicochemical Characterization of CNP Samples

#### 2.2.1. Fourier-Transform Infrared Spectroscopy (FTIR)

Prior to analyzing samples by FTIR, they were freeze dried using a Coolsafe 95-15 PRO freeze drier (ScanVac, Lynge, Denmark) for 48 h to pull out liquid content. FTIR was performed using a Perkin Elmer Spectrum 100 FTIR Spectrometer (Waltham, MA, USA) with a universal attenuated total reflectance (ATR) technique to identify the functional groups in the CNPs. Samples were measured in the range of 280–4000 cm^−1^ at 25 °C. 

#### 2.2.2. Particle Size Distribution (PSD) 

PSD study was performed by using dynamic light scattering (DLS) technique to analyze particle size and their dispersity in the sample. The prepared NPs sample was diluted with distilled water to produce 40% *v*/*v* solution. Then, sample was analyzed by using Zetasizer 3000HSA (Malvern Instruments, Worcestershire, UK). pH of the system was around 6–6.5, near to pKa of chitosan. Each loaded sample (~1000 μL) went through three reading cycles, composed of ~12 measurements to stabilize the sample. Each measurement time was ten seconds. Three different synthesis batches (N = 3) were used in this study to obtain the average particle size and PDI. 

#### 2.2.3. Encapsulation Efficiency (EE) 

The EE was calculated by comparing the difference in absorbance of total compound and free compound. Total compound refers to compound solution only, while free compound refers to the compound that was unencapsulated in CNP-LAA, CNP-TQ, or CNP-LAA-TQ. Both of the solutions must contain the same concentration of compound. The %EE tells of the percentage of compound successfully encapsulated in the carrier; it was calculated using the following formula [41]:

(1) $$\%\mathrm{EE}=\frac{\mathrm{Total}\;\mathrm{compound}\;-\;\mathrm{Free}\;\mathrm{compound}}{\mathrm{Total}\;\mathrm{compound}}\times \;100\%\;$$

The absorbance was measured using UV–VIS spectrophotometer Genesys 10-S (Thermo Fisher Scientific, Waltham, MA, USA) at wavelengths of 244 nm and 254 nm for LAA and TQ, respectively. Triplicate test (N = 3) analysis of single and dual compounds loaded in CNP are reported in the Results and Discussion section. 

#### 2.2.4. Field-Emission Scanning Electron Microscopy (FESEM) 

The surface morphologies of CNP, CNP-LAA, CNP-TQ, and CNP-LAA-TQ were observed using FESEM analysis. Samples were prepared as for DLS study, which were later sonicated for a few seconds. Then, a very small drop of it was put on a cleaned stub and left to dry for three days in a 55 °C oven. The dried sample was coated with gold by using an Emitech K575X sputter coater (Quorum Technologies, West Sussex, UK). Finally, it was analyzed under an electron microscope, FEI NOVA NanoSEM 230 (Thermo Fisher Scientific, Hillsboro, OR, USA). This characterization successfully confirmed the size of the particles as reported in the DLS study. The NP diameters and particle size distributions were calculated using Image J software by National Institutes of Health (version 1.52a) from the FESEM image analysis of 30 individual particles [42].

## 3. Results and Discussion

### 3.1. Determination of Free Functional Groups in Nanoparticle Samples via FTIR

**Figure 4:**
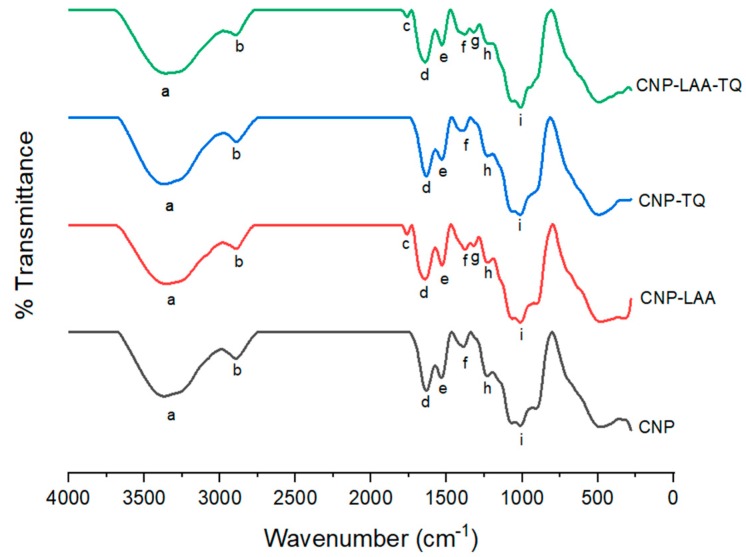
FTIR spectra of CNP, CNP-LAA, CNP-TQ, and CNP-LAA-TQ. Functional groups of all the labelled peaks are stated in Table 1.

FTIR was performed to compare the emerging and losing of functional groups after each component addition, from CNP to final CNP-LAA-TQ formation, as shown in Figure 4. CNP and CNP-TQ both have the same set of peaks. Peak a at 3352 cm^−1^ is the OH stretch, which overlapped with the NH stretch from the primary amine of CS; peak b at 2897 cm^−1^ is the CH stretch; peak d at 1641 cm^−1^ is the amide II carbonyl stretch; peak e at 1532 cm^−1^ is the C=C aromatics contributed by LAA and TQ, which overlapped with NH deformation in CNP; peak f at 1391 cm^−1^ is the CO stretch; peak h at 1237 cm^−1^ is the amide III stretch; and peak i at 1007 cm^−1^ is the CO stretch. CNP-LAA and CNP-LAA-TQ have two additional peaks, which are peaks c and g at 1761 cm^−1^ and 1321 cm^−1^, respectively. Peak c corresponds to the C=O stretch, while peak g resembles the CO stretch. These two peaks might have appeared by the incorporation of LAA.

Each sample can be quantitatively evaluated based on the transmittance percentage, as shown in Table 1. Peaks a, d, e, and i showed comparable values. At peak a, CNP-LAA has a higher reading of OH because LAA has more OH groups as compared to CNP-TQ. The percentage of amide II carbonyl in CNP-LAA-TQ at peak d increased as more C–N–C=O formed. CNP-LAA-TQ also has the highest reading of C=C aromatic rings at peak e, compared to CNP because CNP has no C=C; they emerged from LAA and TQ addition. The presence of both OH and C=C groups at peaks a and e, respectively, had proven that phenolic compounds existed in the sample. Peak I, on the other hand, plays an important role to prove the reaction of inorganic phosphate groups from TPP. CNP shows the highest P=O reading since the TPP has not be fully utilized, while further component (LAA and TQ) addition caused the negatively-charged TPP to be used up. 

CNP was successfully synthesized by looking at the presence of OH, NH_2_, and P=O absorption bands; as seen in CNP spectrum. On the other hand, formation of the encapsulated CNPs (CNP-LAA, CNP-TQ, and CNP-LAA-TQ) can be proven by the appearance of absorption bands of amide II and amide III at peak ~1641 cm^−1^ and ~1237 cm^−1^, respectively, due to the incorporation of compounds containing C=O moieties. The molecular structure of CNP-LAA can be proven by the presence of C=O and OH stretches as shown by the absorption bands at 1761 cm^−1^ and 3352 cm^−1^, respectively. The appearance of absorption bands at 1532 cm^−1^ and 1641 cm^−1^ that are assigned to the C=C and amide II functional groups, respectively, proved the formation of CNP-TQ. As expected, there is the appearance of absorption bands at 3352 cm^−1^ that refers to NH_2_ and OH stretches in CNP-LAA-TQ. In addition, the appearance of the amide III absorption band at 1237 cm^−1^ is the highest in the CNP-LAA-TQ system, which consequently proved the incorporation of both LAA and TQ. 

### 3.2. Particle Size Distribution (PSD) Study by Dynamic Light Scattering (DLS) 

DLS is an analysis of the particles’ movement in solution due to Brownian motion [43]. To be specific, from this study CNP size and polydispersity index, PDI, were obtained. The average size is measured based on the majority of particles’ sizes in a sample. There is no relation between the charge ratio and size reduction, while PDI describes the homogeneity of particles loaded; the lower it is, the more uniform the particles are. The stability of the system is related to PDI value and not affected by zeta potential. Particles with low PDI value have greater stability, while particles with high PDI have lower stability [40]. A sample was prepared as described in the method section and then diluted to 40.0% *v*/*v*. Empty CNPs are expected to show the smallest size with the lowest PDI value. A more significant increment of size and PDI should be seen after the encapsulation of LAA and TQ, depending on the concentration. The size of CNP-LAA, CNP-TQ, and CNP-LAA-TQ increased as a function of LAA or TQ concentration, or both. 

#### 3.2.1. Empty CNP

**Figure 5:**
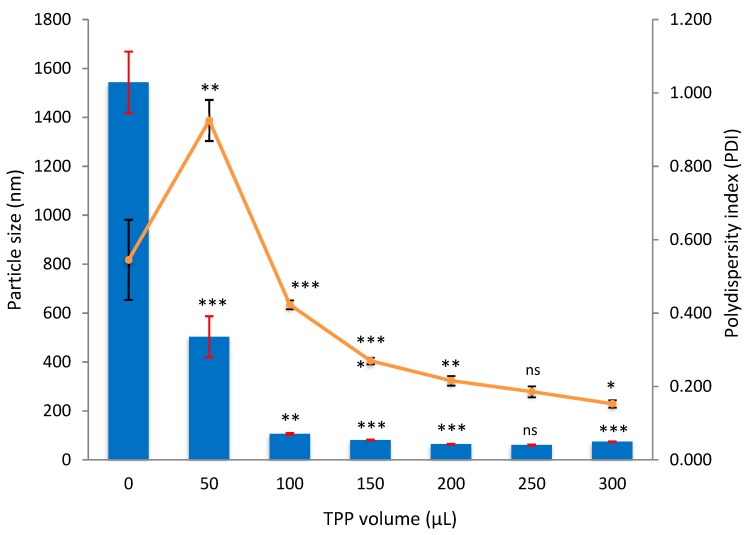
Effect of TPP volume on size and PDI of CNPs. A range of TPP volume, 0 to 300 μL, was added to 600 μL of CS, forming CNPs. The bars represent majority of the particle sizes in a sample, while lines represent the dispersity of particles. *t*-test was conducted to see the significancy of particle size and PDI changes between samples (starts from 0 to 300 μL of TPP). The confidence level was set to 95%. A *p* value of <0.05 shows significant change and labelled as * (more * symbols indicate higher significance), while ns means not significant.

One of the aspects shown in Figure 5 is with respect to changes in particle size as a result of TPP addition in different amounts. At 150 μL of TPP, the particle size started to be less than 100 nm; 80.9 ± 1.5 nm. The size kept on reducing until 300 μL of TPP was utilized; it jumped up to 74.3 ± 1.1 nm. The smallest particle size was 60.5 ± 2.1 nm in which the TPP volume used was 250 μL. The size for empty CNPs was targeted to be around 60–65 nm because after LAA and TQ encapsulation, they will expand more. Another important aspect shown in Figure 5 is polydispersity index (PDI) that refers to homogeneity of a sample. PDI value increased drastically at 50 μL of TPP (with concentration of 0.02 mg/mL), because the chitosan solution arrangement might be disturbed and became too polydisperse when TPP was first loaded. However, a noticeable drop in PDI, 0.925 ± 0.056 to 0.423 ± 0.012, was observed when TPP volume increased from 50 μL to 100 μL. The lowest PDI, 0.153 ± 0.01 was achieved at 300 μL of TPP. Low PDI, less than 0.400, resembles that most of the particles in the sample are about the same size and they are well dispersed.

When using 250 µL TPP to form CNPs, the change in particle size and PDI was not significant (*p*-value > 0.05) compared to 200 µL TPP; which indicated that both volumes of TPP can be used as an optimal parameter to produce CNP. However, 250 µL of TPP was selected to be optimal because it could provide bigger space for LAA to interact. Overall, the optimum volume of TPP needed to make CNPs was 250 μL, based on the smallest particle size and moderate PDI produced, 60.5 ± 2.1 nm and 0.186 ± 0.015, respectively. This TPP volume, 250 μL, was used for the following experiments. 

Empirically, the molar concentrations were not calculated because the molecular weight of chitosan cannot be quantified as it appears to be a chain. However, the concentrations were calculated in mass per volume (mg/mL). Based on Figure 5, the actual CS concentrations after dilution were 0.20, 0.18, 0.17, 0.16, 0.15, 0.14, and 0.13 mg/mL (with increasing volume of TPP). While, the actual concentrations of TPP after dilution were 0, 0.02, 0.04, 0.06, 0.07, 0.08, and 0.09 mg/mL (with increasing volume of TPP).

#### 3.2.2. Single-Loaded CNP

Results for the LAA compound encapsulated in CNP are shown in Figure S1. The smallest CNP-LAA was at 160 μM of LAA with size of 87.3 ± 3.8 nm. However, it did not really differ as compared to empty CNP, which may mean that insufficient LAA was added. The same situation was seen at 310 µM of LAA; not an indicative addition. Meanwhile, 235 μM of LAA showed a significant size increment, implying an adequate amount of LAA inserted; the particle size was 100.4 ± 3.6 nm. For PDI, 160 μM of LAA marked the lowest reading 0.266, followed by 310 µM and 235 µM, with values of 0.277 and 0.333, respectively. 

The PSD results for CNP-TQ are shown in Figure S2. The main difference in particle size and PDI were detected at 150 μM of TQ; both showed lowest readings 74.7 ± 8.5 nm and 0.364 ± 0.097, respectively. The other concentrations presented too much expansion from the empty CNPs, which indicates excessive addition of TQ. As for NPs production, the best product is the one with smallest size but possessed highest encapsulation ability. Size of NPs with hydrophobic component can be reduced by altering the carrier structure. It includes addition of functional group from palmitic acid to CS to increase its hydrophobicity [18]. Then, the size and PDI reduction may be possible because the hydrophobic nature of palmitic acid will ease the linking process of TQ to the carrier. 

#### 3.2.3. Dual-Loaded CNP

**Figure 6:**
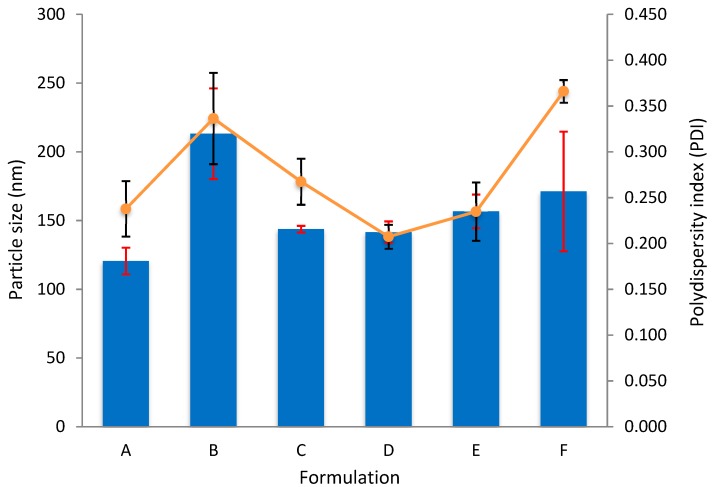
Effect of different sample formulations on particle size and polydispersity index of CNP-LAA-TQ. The bars represent the average particle size in a sample, while lines represent the dispersity of particles.

An exhaustive range of LAA and TQ concentrations had been tested to find the best formulation for dual-loaded CNP, but the ballpark is as shown in Table 2. Six different formulations of LAA and TQ with the same final volume were used to see the changes on size and dispersity, refer to Figure 6 and Table 2. The incorporation of two opposing compounds with different concentrations into CNP will affect the particle size and dispersity. Smallest particle size, 120.6 ± 9.7 nm and the largest particle size, 213.2 ± 33.0 nm, was observed in A and B, respectively. The other compositions resulted in a range of size of around 141 nm to 171 nm. Large particle size may be caused by the insertion of too much of LAA or TQ. As for the PDI, the lowest was 0.207 ± 0.013 and the highest was 0.366 ± 0.012, shown by formulations D and F, respectively. Higher PDI indicates that the sample might have agglomerated. This is possible because CS is very sensitive to water, which can cause it to easily form lumps.

Interaction between hydrophilic LAA and hydrophobic TQ might cause the samples to move differently. All in all, formulation D seems to be convincing as it possesed the lowest PDI, 0.207 ± 0.013, with moderate particle size, 141.5 ± 7.8 nm. Nonetheless, it may not be the best formulation as other characterizations have to be considered too. 

#### 3.2.4. Comparison in Particle Size and Dispersity Between Empty, Single-Loaded and Dual-Loaded CNP

**Figure 7:**
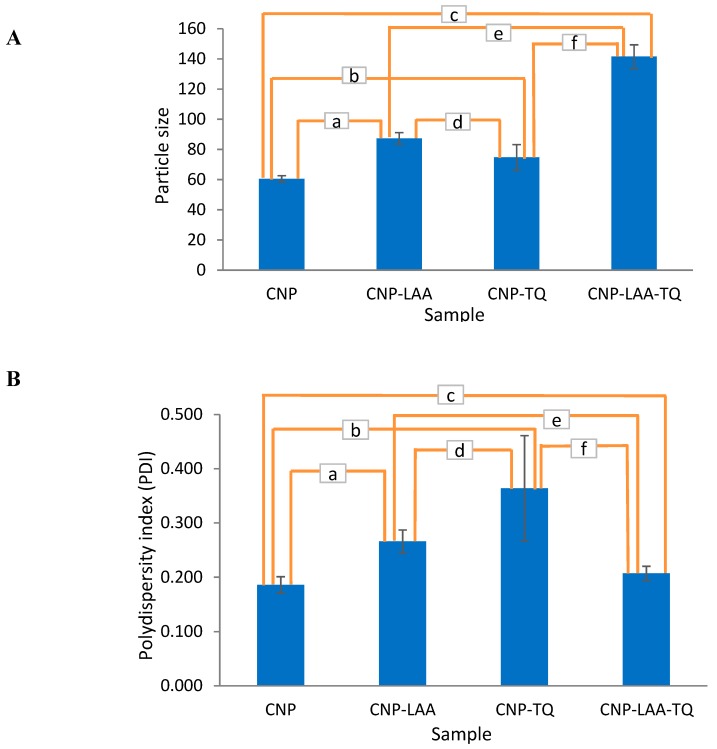
(**A**) Particle size of CNP, CNP-LAA, CNP-TQ, and CNP-LAA-TQ, with *t*-test conducted to see the significancy of particle size changes between samples. The confidence level was set to 95% and the results are labelled as a to f. (a) Significant *** with a *p* value of 0.0004, (b) significant * with a *p* value of 0.0468, (c) significant **** with a *p* value of <0.0001, (d) not significant with a *p* value of 0.079, (e) significant *** with a *p* value of 0.0004, and (f) significant *** with a *p* value of 0.0006. (**B**) Polydispersity index and significancy test (*t*-test) of CNP, CNP-LAA, CNP-TQ, and CNP-LAA-TQ. (a) Significant ** with a *p* value of 0.0057, (b) significant with a *p* value of 0.0349, (c) not significant with a *p* value of 0.1340, (d) not significant with a *p* value of 0.1623, (e) significant * with a *p* value of 0.0147, and (f) not significant with a *p* value of 0.0504.

As seen in Figure 7A, each compound addition resulted in the increment of particle size. CNP size widened to 44.4% and 23.6% after separate encapsulation of LAA and TQ, respectively. Dual-loaded CNP, CNP-LAA-TQ, on the other hand showed more than twice the addition in size compared to CNP. This is contributed by the existence of more complex interactions between compounds and the carrier. CNP-TQ presented a higher increment in size, 89.4%, when LAA was loaded into the system, while CNP-LAA size increased to 62.2% after the incorporation of TQ into it. Figure 7B is describing the changes in polydispersity (PDI) of particles in all samples. For the encapsulated CNP, the dual-loaded version has the smallest PDI reading, 0.207 as compared to any of the single-loaded CNP. This may tell that TQ and LAA could form better uniformity when placed together. CNP-TQ showed highest PDI because of the hydrophobicity of TQ. It may have created uneven barriers within the hydrophilic components, consequently, creating less uniform particles. 

### 3.3. Encapsulation Efficiency (EE) Study of Nanoparticle Samples 

Physical aspects of nanoparticles either encapsulated or not were reported above in terms of particle size and dispersity. Encapsulation efficiency (EE) is a correlation to them, which tells about the ability of each component, LAA, and TQ to stay in CNPs carrier. Their presence in the sample were detected at specific wavelength; LAA at 244 nm and TQ at 254 nm [44,45,46]. EE was measured by a UV–VIS spectrophotometer that detects the amount of light absorbed by a sample at their particular wavelength and displays them in absorbance. Two-stage EE analysis was performed; first by using single-loaded compound-NPs (CNP-LAA and CNP-TQ), and second by using dual-loaded compounds-NPs (CNP-LAA-TQ).

#### 3.3.1. Single-Loaded CNP 

Figure S3 shows percent encapsulation of LAA in CNP-LAA. The lowest concentration of LAA, 160 µM, was efficiently entrapped in CNPs, with the highest EE reading of 69.3 ± 1.8%. An increased amount of LAA reduced the EE because it might be too much and caused it to drive out of the carrier. A total of 235 µM showed 64.2 ± 2.8 %EE, while 310 µM produced 61.1 ± 1.2 %EE. Meanwhile, Figure S4 displays the percent encapsulation of TQ in CNP-TQ. The incorporation of 150 µM TQ into CNPs resulted in the highest encapsualtion percentage, 68.7 ± 4.8%. This means that more than 60% of the loaded TQ had successfully been encapsuated. The second-best concentration was 200 µM (59.0 ± 17.3%), followed by 100 µM (37.6 ± 6.4%). 

#### 3.3.2. Dual-Loaded CNP 

The set of TQ concentrations used for EE detection in dual encapsulation CNPs was narrowed to only 100 µM and 150 µM. The highest concentration 200 µM was eliminated because it was excessive and unnecessary based on TQ single-encapsulation results. Figure S5 depicts the percent encapsulation of LAA in dual-loaded CNP. Sample C that was made up of 310 µM of LAA and 100 µM of TQ displayed the highest EE 73.5 ± 2.4%, followed by sample A and B, 47.9 ± 5.7% and 31.8 ± 2.8%, respectively. D, F and E on the other hand were the lowest three samples; 22.8 ± 3.2%, 21.6 ± 4.3% and 11.1 ± 3.6%, respectively. Although C had highest EE, it could also mean that TQ was not well encapsulated as CNPs had almost been filled with LAA. In addition, for single LAA encapsulation, 310 µM of LAA showed the lowest EE reading. 

Therefore, EE of TQ in dual-loaded CNP was performed to select the best formulation and the outcomes are shown in Figure S6. TQ concentration of 150 µM (D, E, F) presented higher EE as compared to 100 µM (A, B, C), as seen in Figure S6. By increasing 50 µM of TQ, the EE increased more than twice. D that possessed 150 µM of TQ with 160 µM of LAA had the most satisfying percent of encapsulation (35.6 ± 3.6%), which describes an ideal setting that helped it to stay in the carrier. Overall results for dual-loaded CNP is shown in Table 3.

#### 3.3.3. Comparison in EE between Single-Loaded and Dual-Loaded CNP

**Figure 8:**
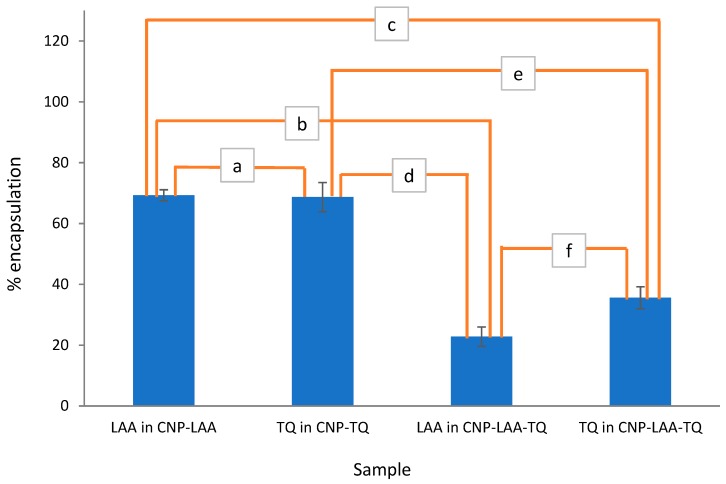
Percent encapsulation of LAA in CNP-LAA, TQ in CNP-TQ, LAA in CNP-LAA-TQ, and TQ in CNP-LAA-TQ. A *t*-test was conducted to see the significance of percent encapsulation changes between samples. The confidence level was set to 95% and the results are labelled as a to f. (a) Not significant with a *p* value of 0.8662, (b) significant **** with a *p* value of <0.0001, (c) significant **** with *p* value of <0.0001, (d) significant *** with a *p* value of 0.0002, (e) significant *** with a *p* value of 0.0002, and (f) significant * with *p* value of 0.0334.

The percentage of compound encapsulation into all CNP samples is shown in Figure 8. Both single-loaded CNP displayed quite the same amount of EE, approximately 69.0%. However, the capability of two compounds to be loaded together decreased to almost half. LAA that stayed in dual-loaded CNP has lower % encapsulation, 22.8%, as compared to TQ in dual-loaded CNP, 35.6%. It may be caused by the flow of synthesizing dual-loaded CNP, as shown in Figure 3. TQ was added first to CS, followed by LAA which later being added together with TPP. TQ might have conquered more space in the CNP, leaving a small area for LAA.

### 3.4. Morphological Study of Nanoparticle Samples by FESEM 

**Figure 9:**
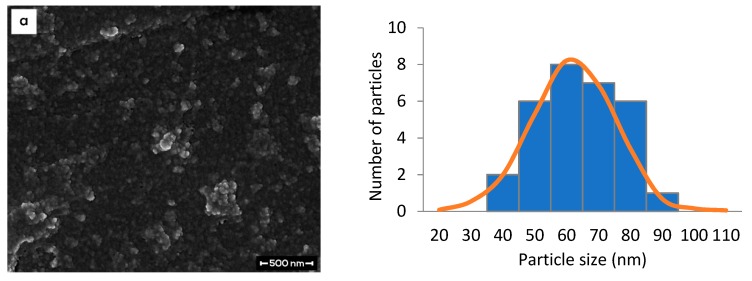

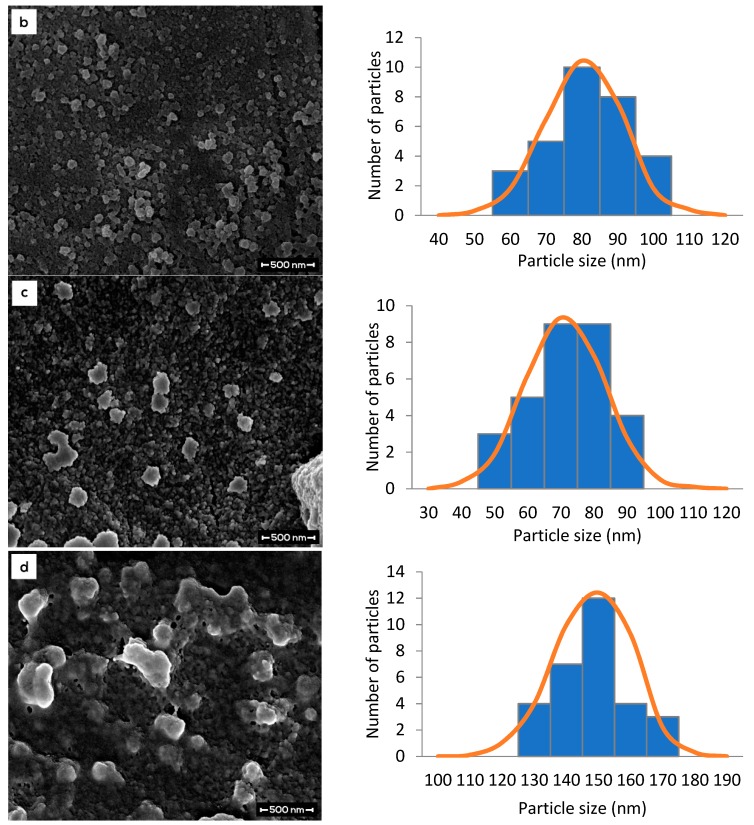
Surface morphology (**left**) and particle size distribution (**right**) of (**a**) CNP, (**b**) CNP-LAA, (**c**) CNP-TQ, and (**d**) CNP-LAA-TQ. The particle size from FESEM results match the trend in the PSD study.

Figure 9 shows the morphology of nanoparticles at 100,000× magnification. The largest particle was found in dual-loaded CNP (image d), while the smallest particle was found in empty CNP (image a). Sequential addition of each component resulted in an increase in the size of particles, which correlated with PSD analysis. From CNP to CNP-LAA (image b) the majority of particle size increased from around 60.0 nm to around 80.0 nm (33.3% increase). The difference indicated that LAA was successfully encapsulated into CNP. Once TQ was added into CNP, the size increased to around 70.0 to 80.0 nm (image c). The particle size after addition of both LAA and TQ into CNP was around 150.0 nm (image d), which is twice compared to single-loaded particles (CNP-LAA and CNP-TQ).

Particle size analyzed by both PSD and FESEM were in range. The average FESEM particle size in Figure 9 can be compared with PSD particle size in Table 4. PSD showed larger particle sizes for CNP and CNP-LAA, with a difference of 0.8% (0.5 nm) and 9.1% (7.3 nm), respectively. While, CNP-TQ and CNP-LAA-TQ displayed bigger particle size when analyzed by FESEM, with a difference of 0.4% (0.3 nm) and 6.0% (8.5 nm), respectively. The particle size of samples c and d analyzed by FESEM were slightly larger as compared to the PSD analysis, due to agglomeration and gold coating. Overall, the samples images are expected to show discrete spherical NPs. However, agglomeration appeared, as seen in images a and d, due to the characteristic of CS that can easily aggregate in the presence of water [47]. A study by Khlibsuwan, et al suggested that ethanol may assist in decreasing the swelling effect of nanocomposites [47]. The problem can also be encountered by lengthening the centrifugation time and shortening the sonication time in preparing the sample.

### 3.5. Formulation Selection of Single- and Dual-Loaded CNP

From PSD study, the best TPP volume was 250 µL, with concentration of LAA and TQ of 160 µL and 150 µL, respectively. This combination produced CNP-LAA-TQ with size of 141.5 ± 7.8 nm and a PDI value of 0.207 ± 0.013 (Sample D, Table 2). EE study, on the other hand, further strengthens the selection of optimum formulation. For single-loaded CNP, the highest EE of LAA was 69.3 ± 1.8%, at concentration of 160 µM, while highest EE of TQ was 68.7 ± 4.8%, at concentration of 150 µM. From those data, the optimum concentration of LAA and TQ are expected to be 160 µM and 150 µM, respectively, to give the highest EE for dual-loaded CNP. This concentration combination was labelled as Sample D in Table 3. Based on Table 3, the EE of LAA in sample D was quite low, 22.8 ± 3.2%, as compared to sample C with highest EE of 73.5 ± 2.4%. It may be caused by higher content of LAA in sample C which is almost double that of sample D. However, the expectation was achieved in analyzing EE of TQ in CNP-LAA-TQ. Comparing the EE of TQ in CNP-LAA-TQ, sample D showed the highest EE of 35.6 ± 3.6%. Based on data from all study sections, an optimum formulation for CNP-LAA-TQ was found to be 250 µL of TPP, 160 µM of LAA, and 150 µL of TPP. The summary of the optimum results from PSD and EE is shown in Table 4. 

Outcomes from this study are hoped to be beneficial for pharmaceutical and medical fields. Through further modification, %EE can be elevated for better drugs contain. This study would be extended to discover the possibility of dual drug-encapsulated NPs for transdermal application; delivering drugs with ease and free of pain. 

## 4. Conclusions

CNP-LAA-TQ was successfully synthesized via ionic gelation method with drugs: CS volume ratio of ~1:2. Few sets of LAA and TQ concentration were tested and the best formulations were 160 µM and 150 µM, respectively. The selection was made based on the most optimum particle size, PDI and %EE, which were 141.5 ± 7.8 nm, 0.207 ± 0.013 and 22.8 ± 3.2%, 35.6 ± 3.6%, respectively. These small spherical and moderately-dispersed NPs, with good encapsulation efficiency of LAA and TQ, act as a potentially remarkable development of dual-compound encapsulation of CNPs. This indirectly widens the prospect for NP study, focusing on combining different classes of drugs—hydrophilic and hydrophobic—together. In a larger view, this study provides a better solution for illness treatment that requires more than one drug action in a single effective carrier.

Drug release study of LAA and TQ will be further explored in vitro by using biological liquids, such as phosphate buffered saline (PBS), serum-containing media, such as DMEM supplemented with fetal bovine serum (FBS), and PenStrep.

## Figures and Tables

**Table 1:** Transmittance percentage of the functional groups in CNP, CNP-LAA, CNP-TQ, and CNP-LAA-TQ.

	% Transmittance								
Peak	a	b	c	d	e	f	g	h	i
Functional Group	NH_2_ Amine I, OH Stretch	CH Stretch	C=O Stretch	CN–C=O Amide II carbonyl	C=C Stretch, NH Deformation	CO Stretch	CO Stretch	CN Amide III Stretch	P=O Stretch
Wavenumber (cm^−1^)	3352	2897	1761	1641	1532	1391	1321	1237	1007
CNP	38.7	74.6	None	42.2	56.3	84.2	None	56.8	13.1
CNP-LAA	42.2	75.0	90.3	47.4	60.0	72.9	78.3	62.0	8.1
CNP-TQ	33.9	75.2	None	47.2	58.0	86.0	None	61.1	8.1
CNP-LAA-TQ	38.4	73.4	88.1	49.4	64.4	74.7	76.2	67.5	8.2

**Table 2:** Particle size and PDI of CNP-LAA-TQ according to LAA and TQ concentration.

Formulation	Concentration (μM)		Particle Size (nm ± sd)	Polydispersity Index (±sd)
	LAA	TQ		
A	160	100	120.6 ± 9.7	0.238 ± 0.030
B	235	100	213.2 ± 33.0	0.336 ± 0.050
C	310	100	143.7 ± 2.4	0.267 ± 0.025
D	160	150	141.5 ± 7.8	0.207 ± 0.013
E	235	150	156.6 ± 12.2	0.235 ± 0.032
F	310	150	171.2 ± 43.5	0.366 ± 0.012

**Table 3:** Percent encapsulation efficiency of LAA and TQ in CNP-LAA-TQ sample.

Sample	Initial (μM)		EE of		Loading (μM)	
	LAA	TQ	LAA in CNP-LAA-TQ (% ± sd)	TQ in CNP-LAA-TQ (% ± sd)	LAA	TQ
A	160	100	47.9 ± 5.7	17.8 ± 3.1	76.6	17.8
B	235	100	31.8 ± 2.8	12.8 ± 9.6	74.7	12.8
C	310	100	73.5 ± 2.4	13.3 ± 2.8	227.9	73.5
D	160	150	22.8 ± 3.2	35.6 ± 3.6	36.5	53.4
E	235	150	11.1 ± 3.6	30.3 ± 9.0	26.1	45.5
F	310	150	21.6 ± 4.3	31.6 ± 7.0	67.0	47.4

**Table 4:** Summary of best results from both PSD and EE studies. Differences in particle size, PDI, and %EE are listed below to highlight the highest achieved values.

Type of CNP	PSD		%EE
	Particle Size (nm)	PDI	
Empty	60.5 ± 2.1	0.186 ± 0.015	*nr
Single-loaded LAA	87.3 ± 3.8	0.266 ± 0.021	69.3 ± 1.8
TQ	74.7 ± 8.5	0.364 ± 0.097	68.7 ± 4.8
Dual-loaded LAA	141.5 ± 7.8 (D)	0.207 ± 0.013 (D)	22.8 ± 3.2 (D)
TQ			35.6 ± 3.6 (D)

*nr = not related.

## Supplementary Materials

The following are available online at http://www.mdpi.com/2079-4991/8/11/920/s1, Figure S1: Effect of LAA concentration on particle size and PDI of CNP-LAA. LAA was dissolved in TPP solution prior to addition into CS solution. The bars represent majority of particle size in a sample, while lines represent dispersity of particles, Figure S2: Effect of TQ concentration on particle size and PDI index of CNP-TQ. TQ was first dissolved in 99 % DMSO, but after few times of dilution, DMSO final concentration was 4.17 %. The bars represent majority of particle size in a sample, while lines represent dispersity of particles, Figure S3: Percent encapsulation of LAA in CNP-LAA, Figure S4: Percent encapsulation of TQ in CNP-TQ, Figure S5: Percent encapsulation of LAA in CNP-LAA-TQ and Figure S5: Percent encapsulation of LAA in CNP-LAA-TQ, and Figure S6: Percent encapsulation of TQ in CNP-LAA-TQ.
