# Supplementary material for: Synthesis and Optimization of Chitosan Nanoparticles Loaded with l-Ascorbic Acid and Thymoquinone
Source: Nanomaterials (Basel). 2018 Nov 7;8(11):920. doi: 10.3390/nano8110920 (PMC6267081; doi:10.3390/nano8110920)
Supplement: Supplementary file 1 [file nanomaterials-08-00920-s001.pdf]

**Supplementary Materials**

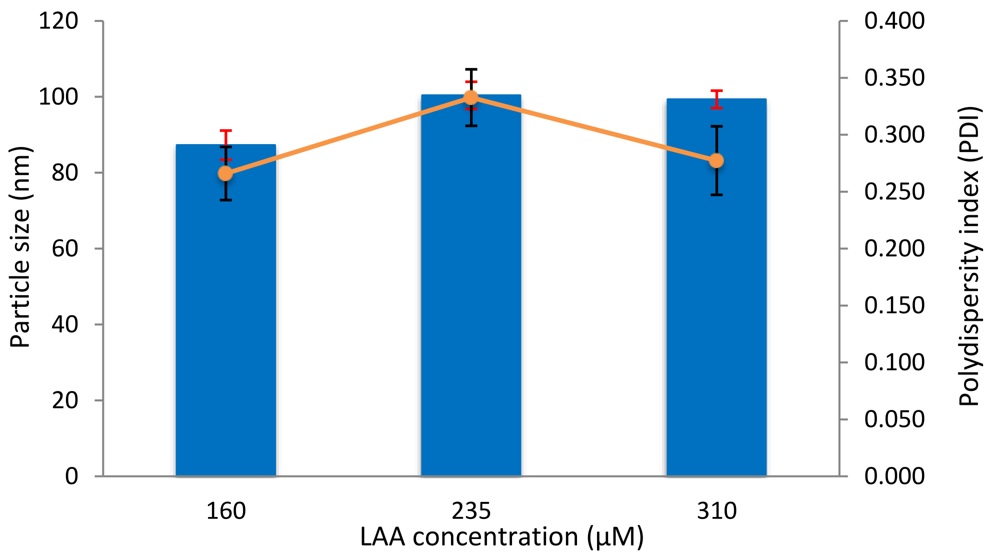


**Figure S1:** Effect of LAA concentration on particle size and PDI of CNP-LAA. LAA was dissolved in TPP solution prior to addition into CS solution. The bars represent majority of particle size in a sample, while lines represent dispersity of particles.

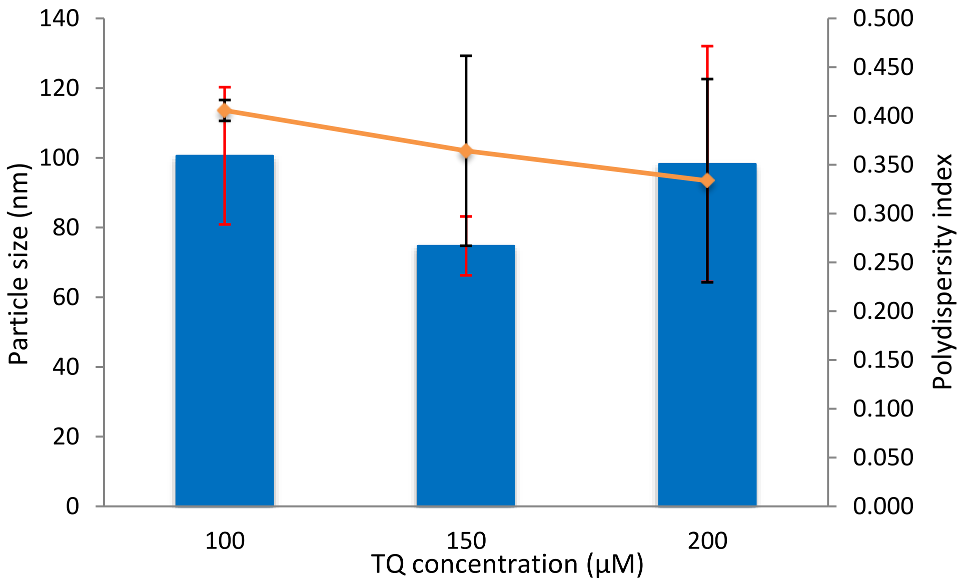


**Figure S2:** Effect of TQ concentration on particle size and PDI index of CNP-TQ. TQ was first dissolved in 99 % DMSO, but after few times of dilution, DMSO final concentration was 4.17 %. The bars represent majority of particle size in a sample, while lines represent dispersity of particles.

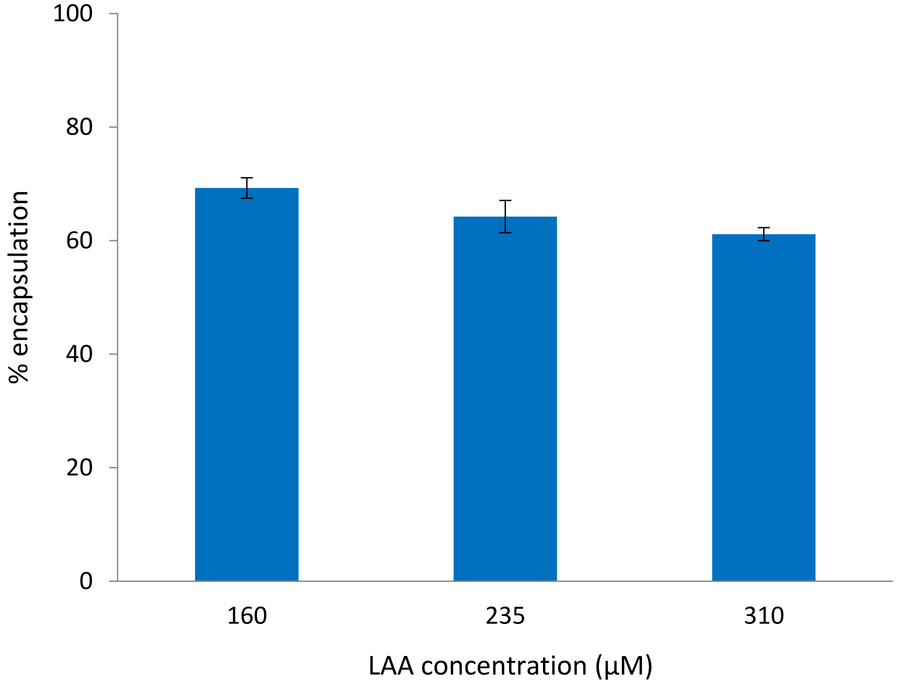


**Figure S3:** Percent encapsulation of LAA in CNP-LAA.

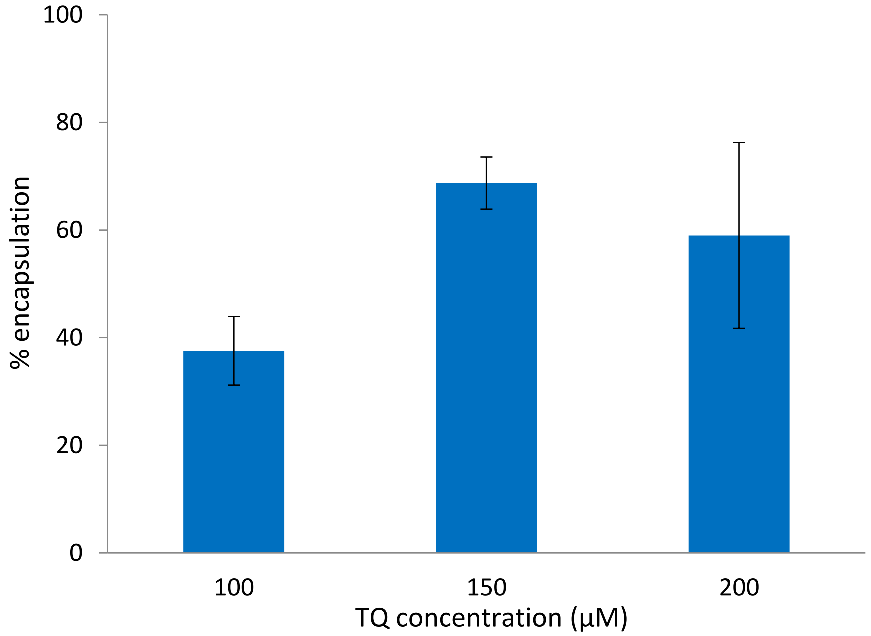


**Figure S4:** Percent encapsulation of TQ in CNP-TQ.

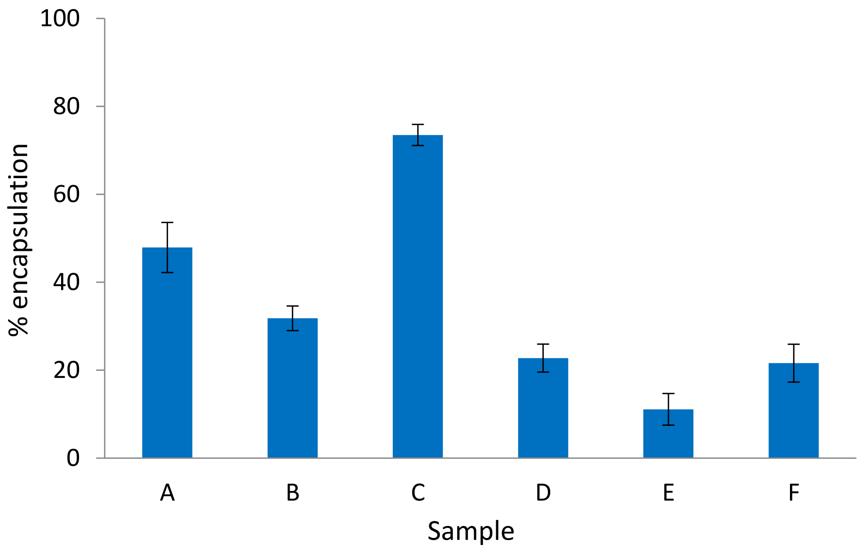


**Figure S5:** Percent encapsulation of LAA in CNP-LAA-TQ.

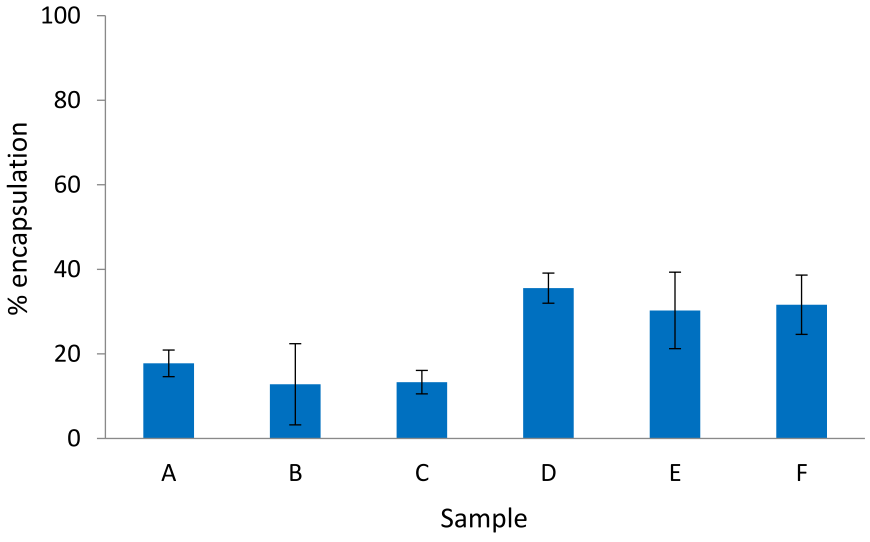


**Figure S6:** Percent encapsulation of TQ in CNP-LAA-TQ.

**Chemical structures**

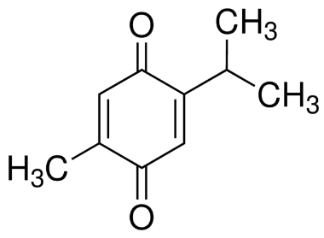


Thymoquinone

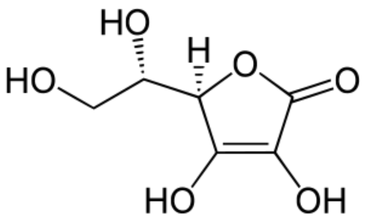


L-ascorbic acid

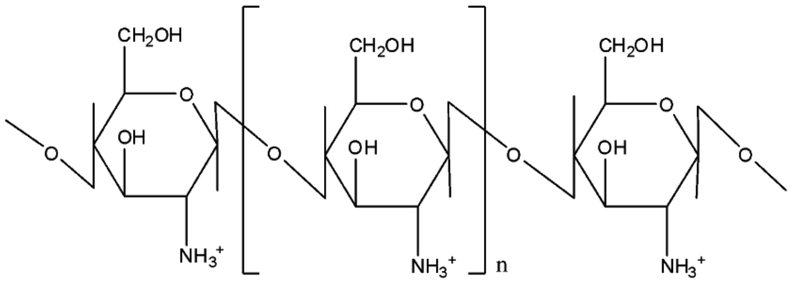


Chitosan

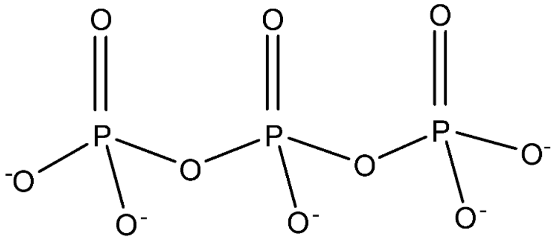


Tripolyphosphate
